# Supplementary material for: Characteristics of the Genome, Transcriptome and Ganoderic Acid of the Medicinal Fungus Ganoderma lingzhi
Source: J Fungi (Basel). 2022 Nov 28;8(12):1257. doi: 10.3390/jof8121257 (PMC9784716; doi:10.3390/jof8121257)

Compound 1

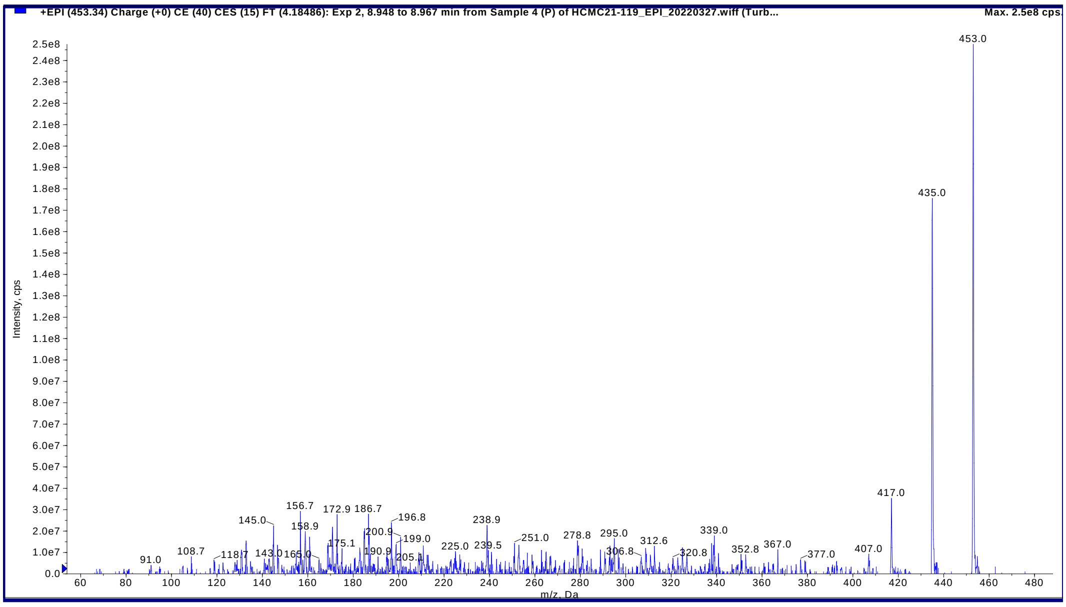

Compound 2

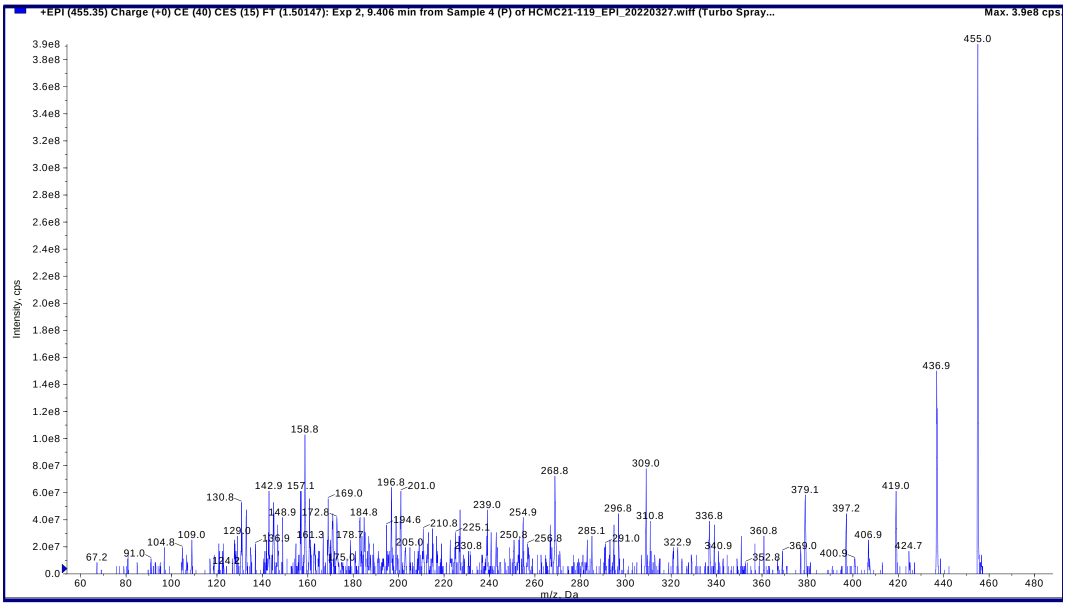

Compound 3

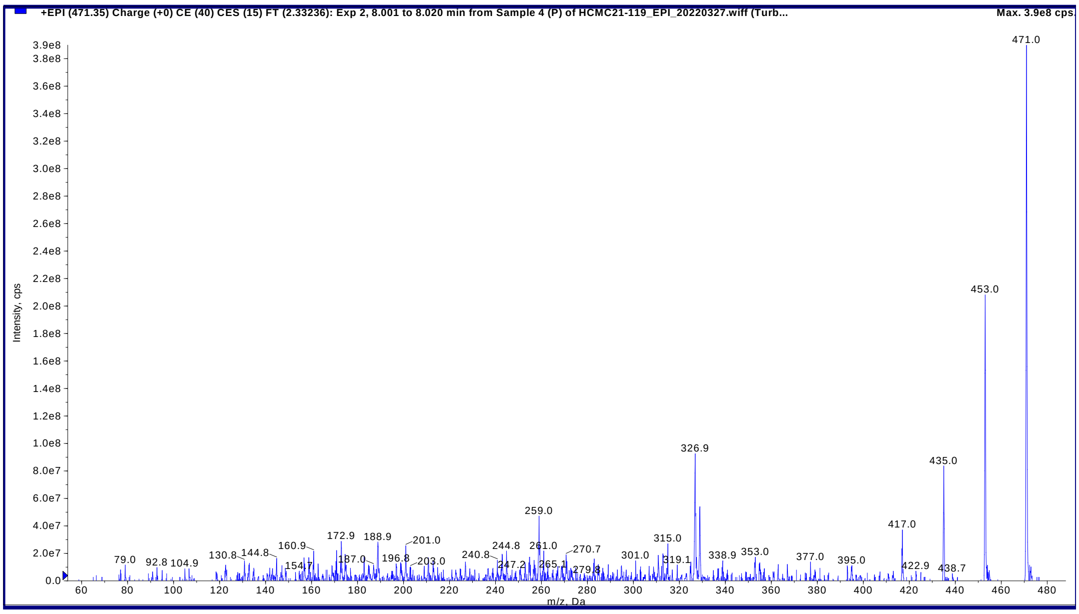

Compound 4

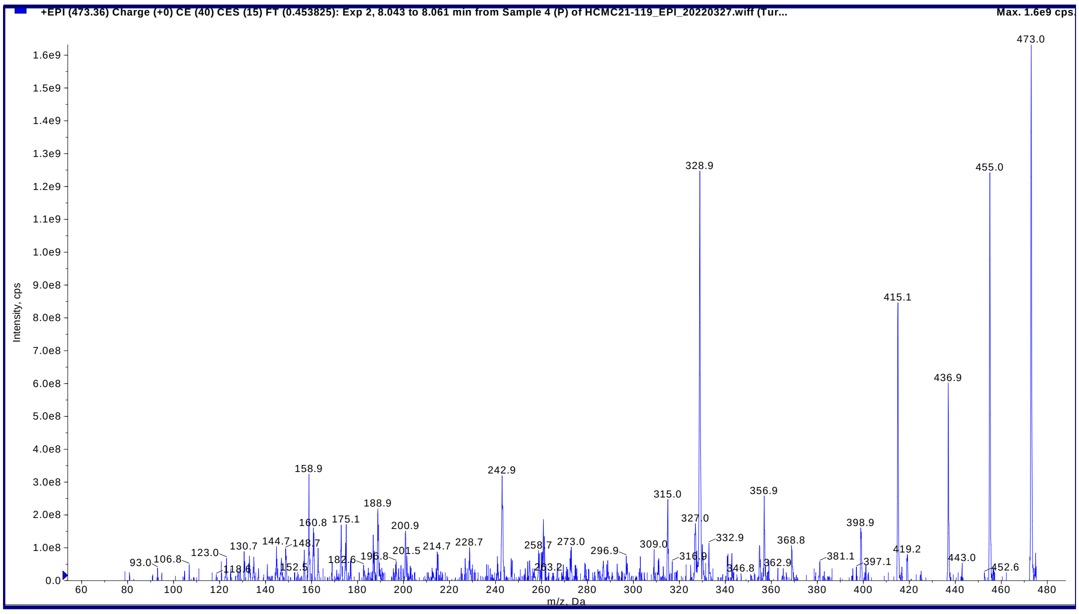

Compound 5

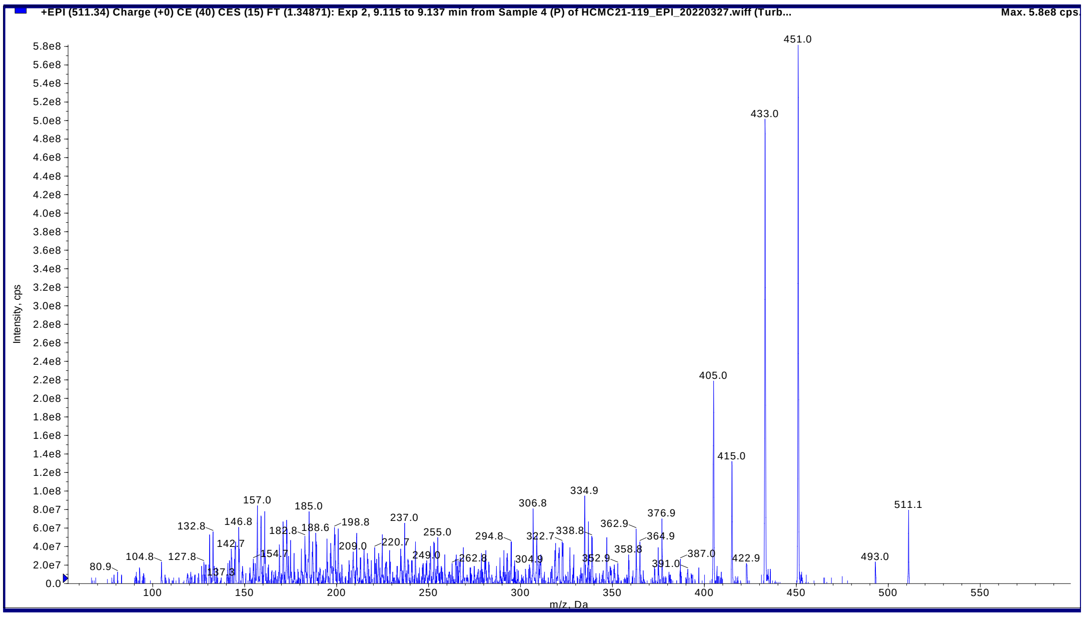

Compound 6

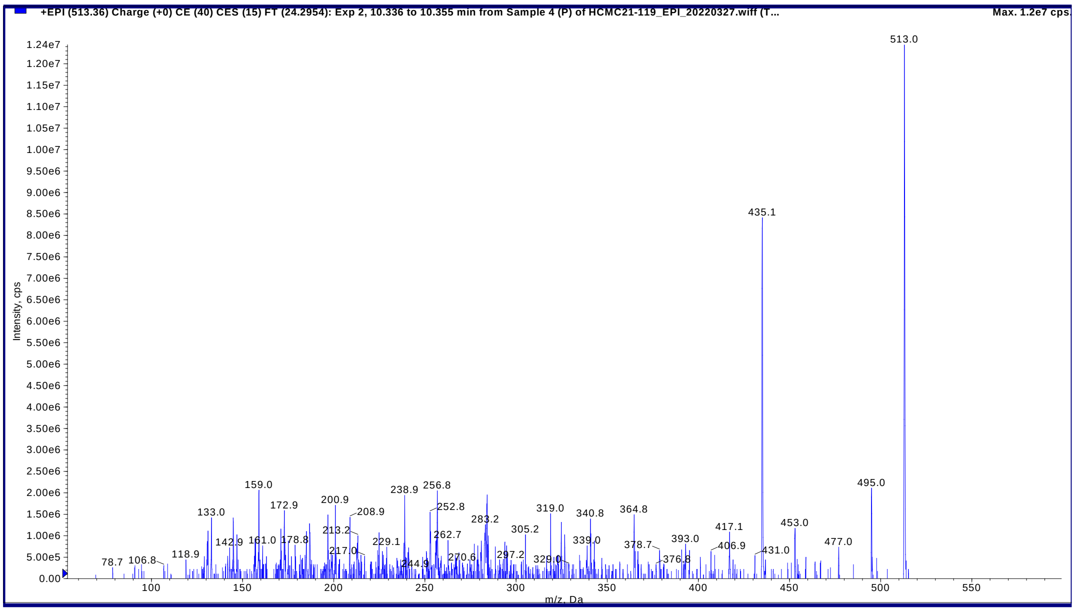

Compound 7

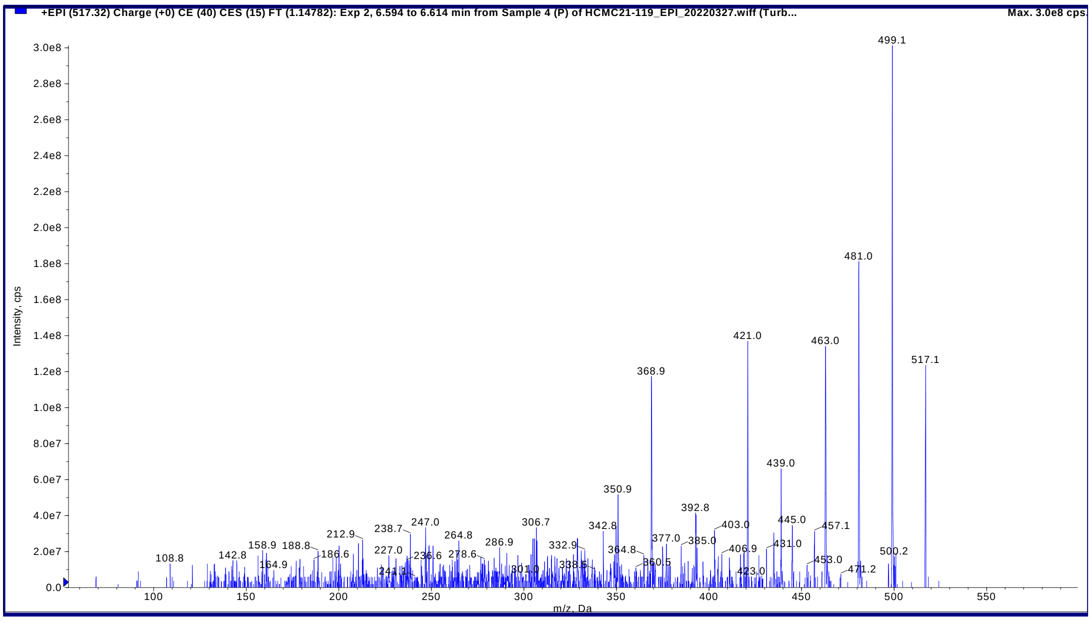

Compound 8

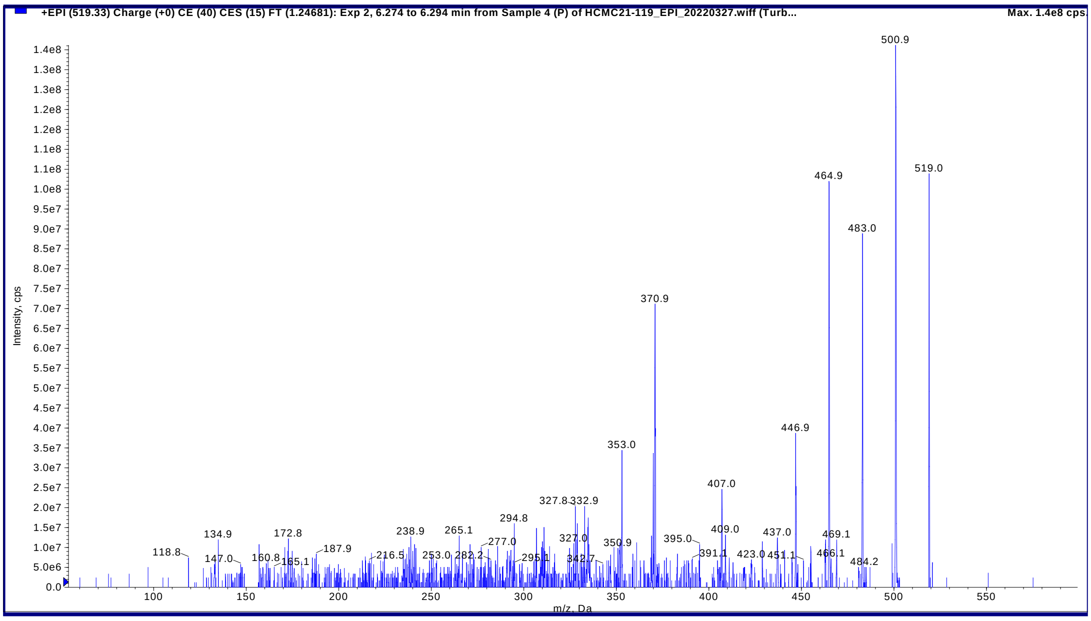

Compound 9

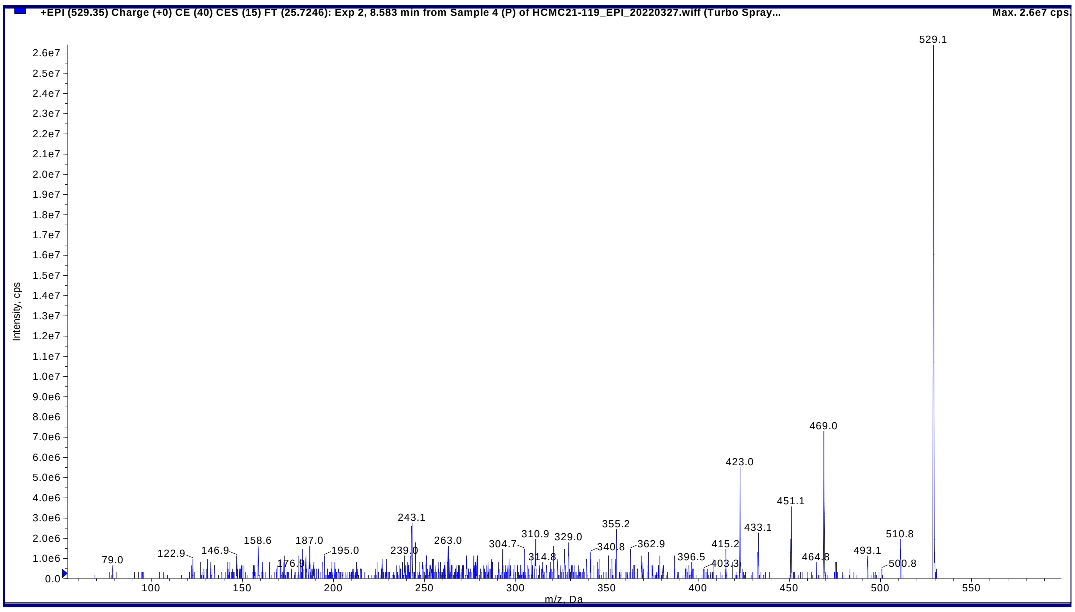

Compound 10

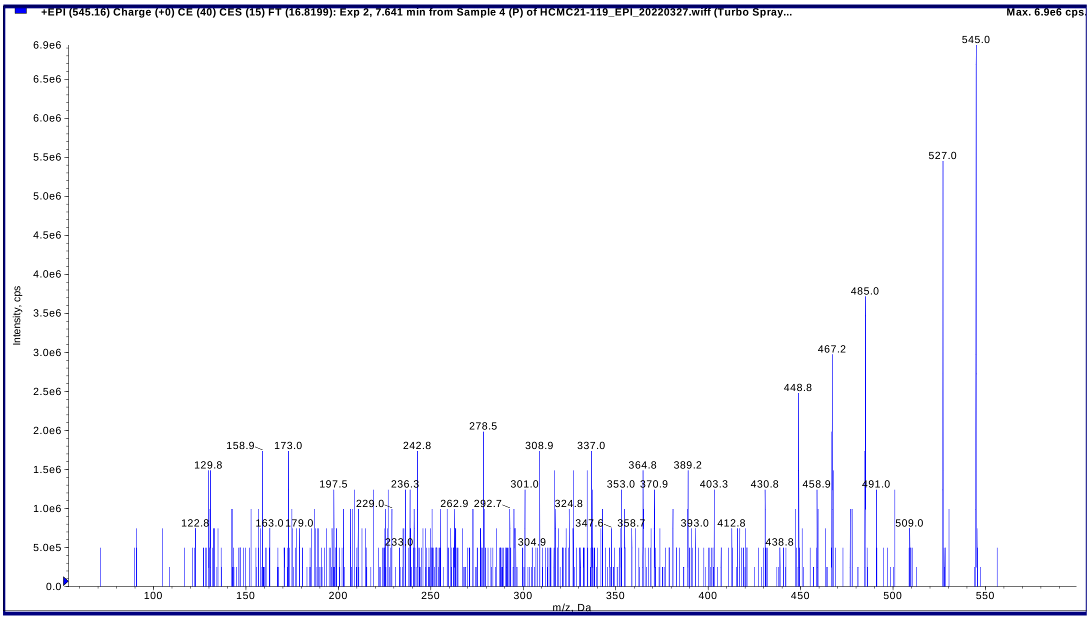

Compound 11

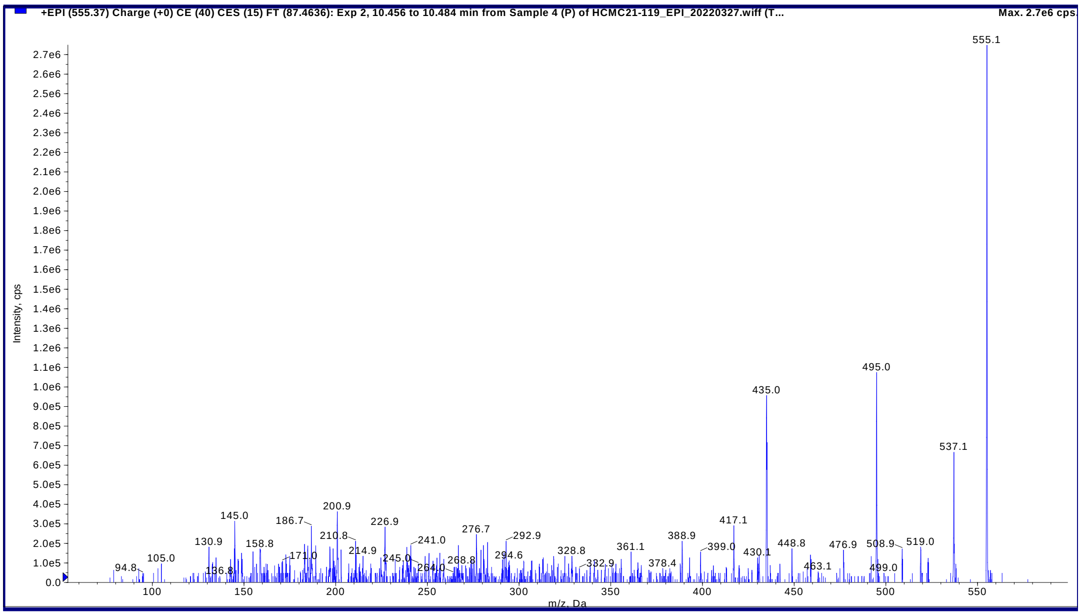

Compound 12

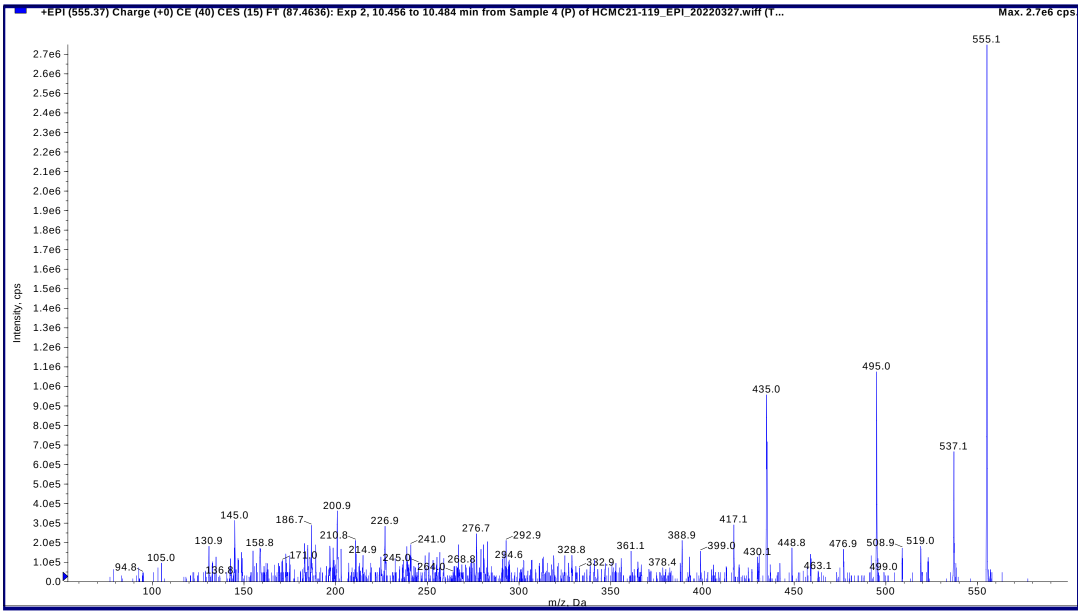

Compound 13

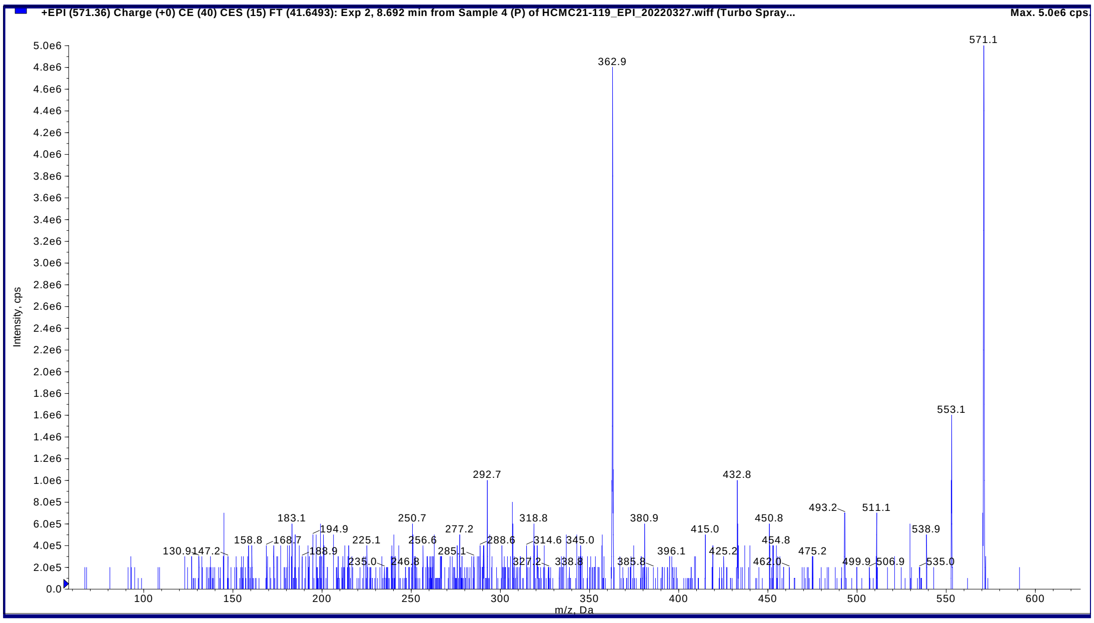

Compound 14

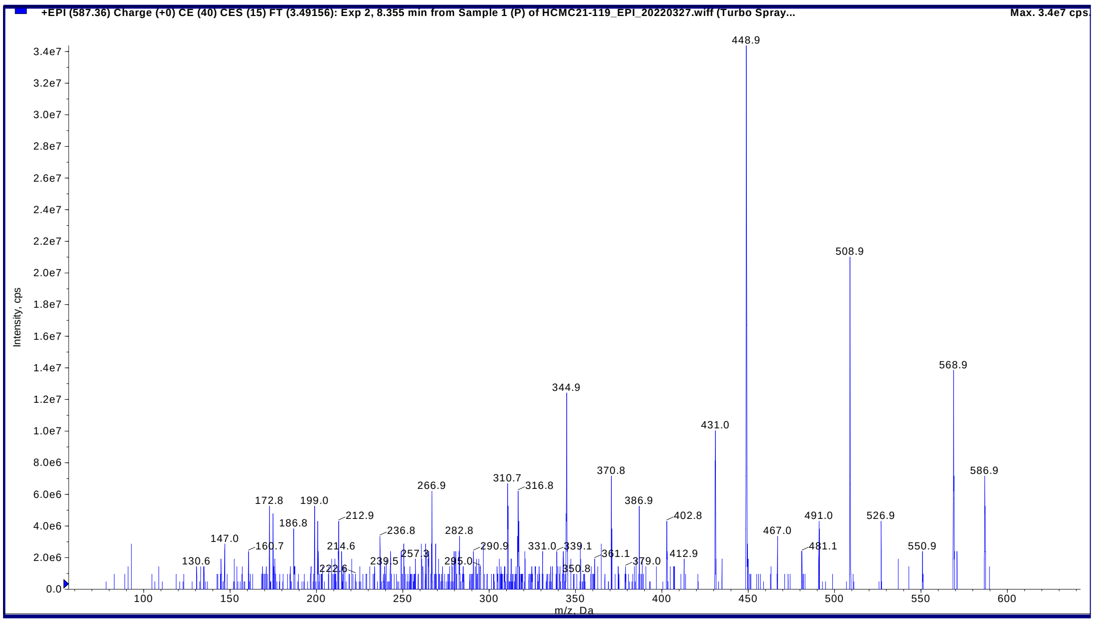

Compound 15

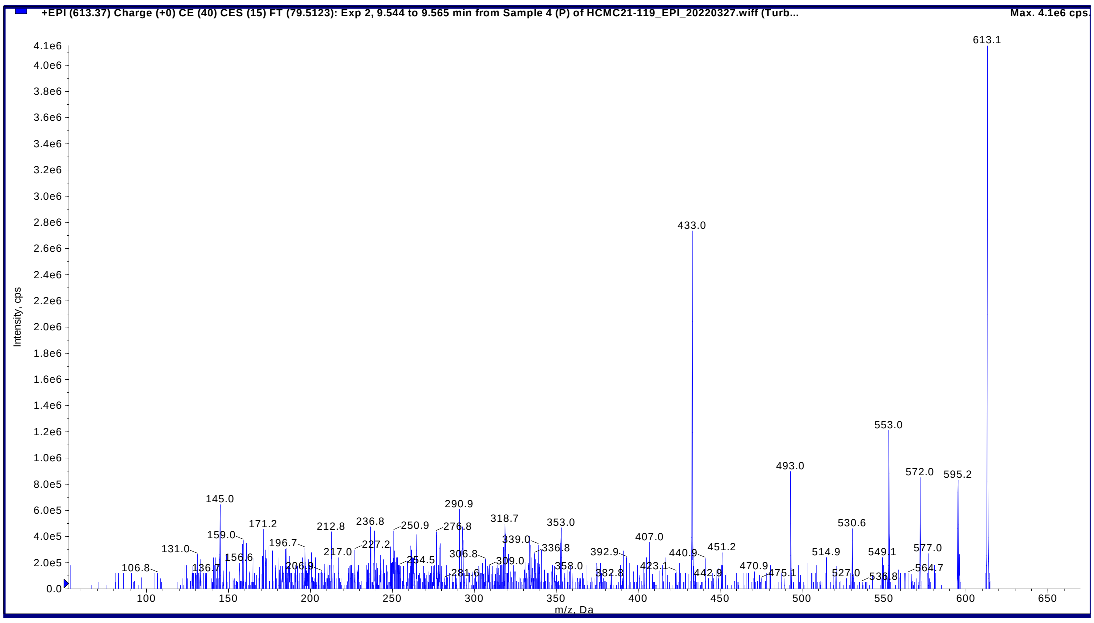

Compound 16

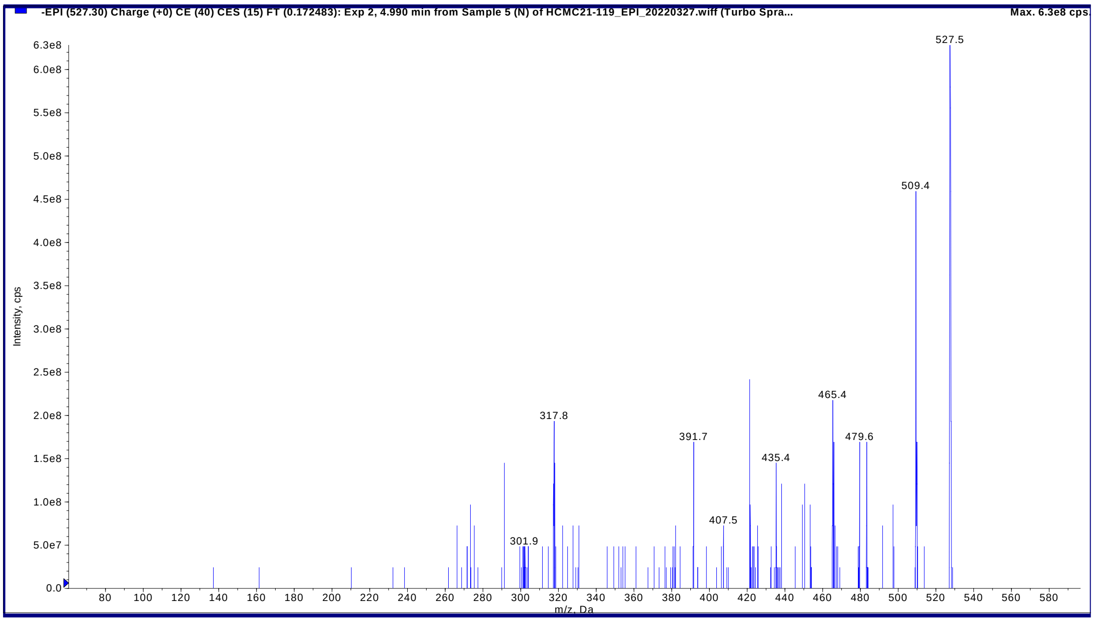

Compound 17

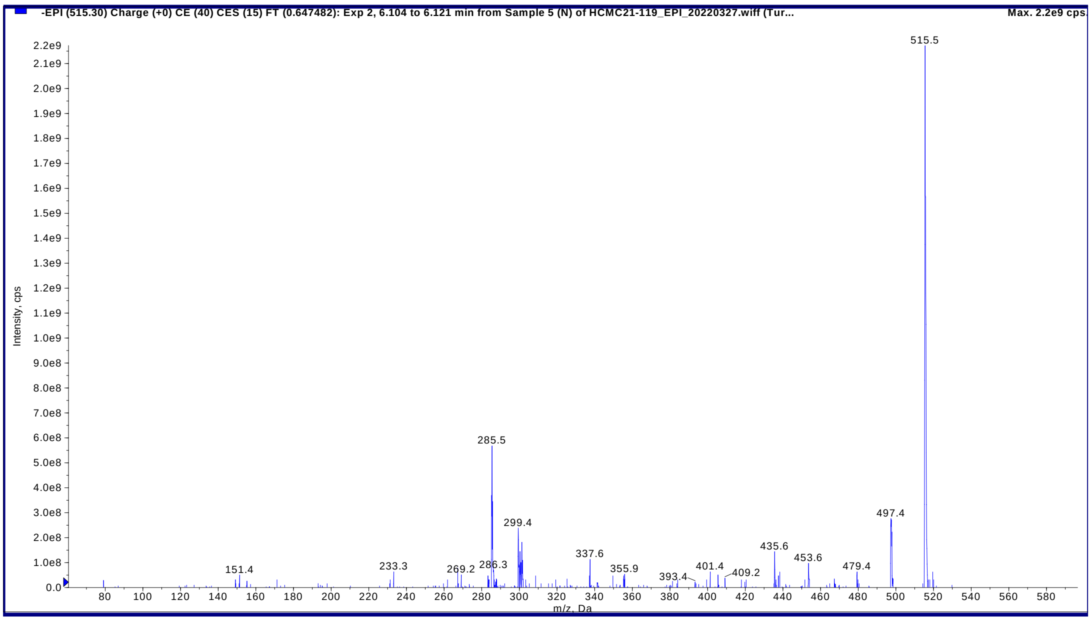

Compound 18

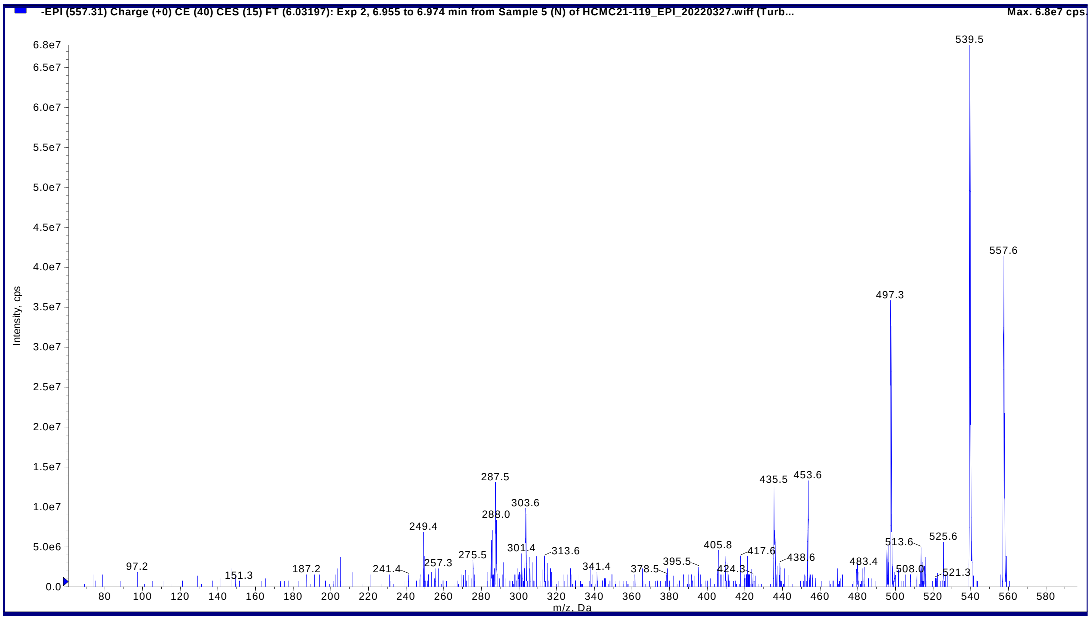

Compound 19

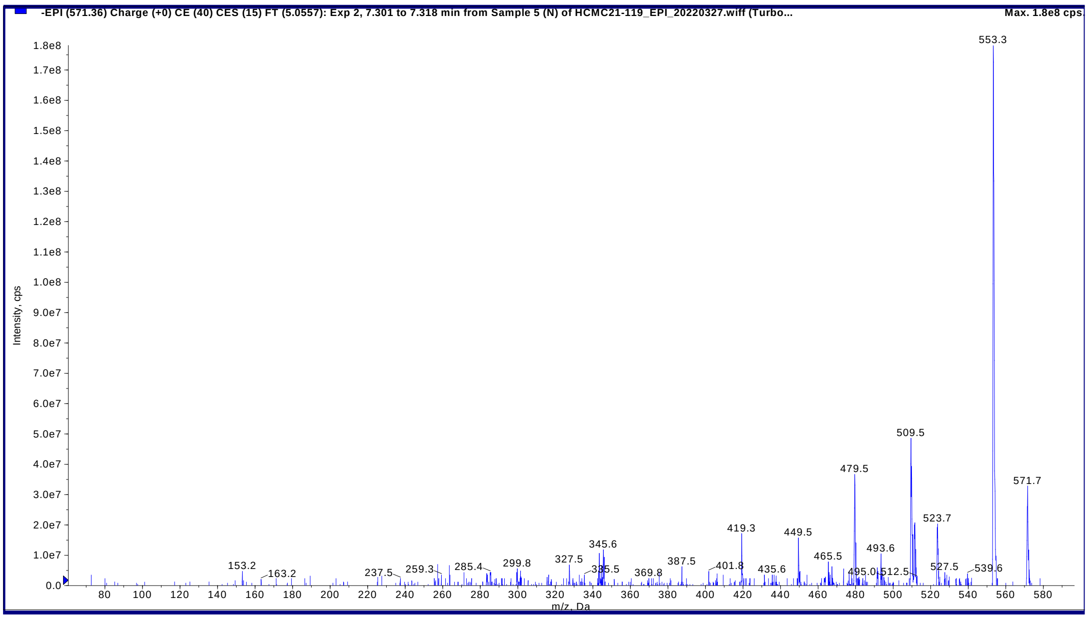

Compound 20

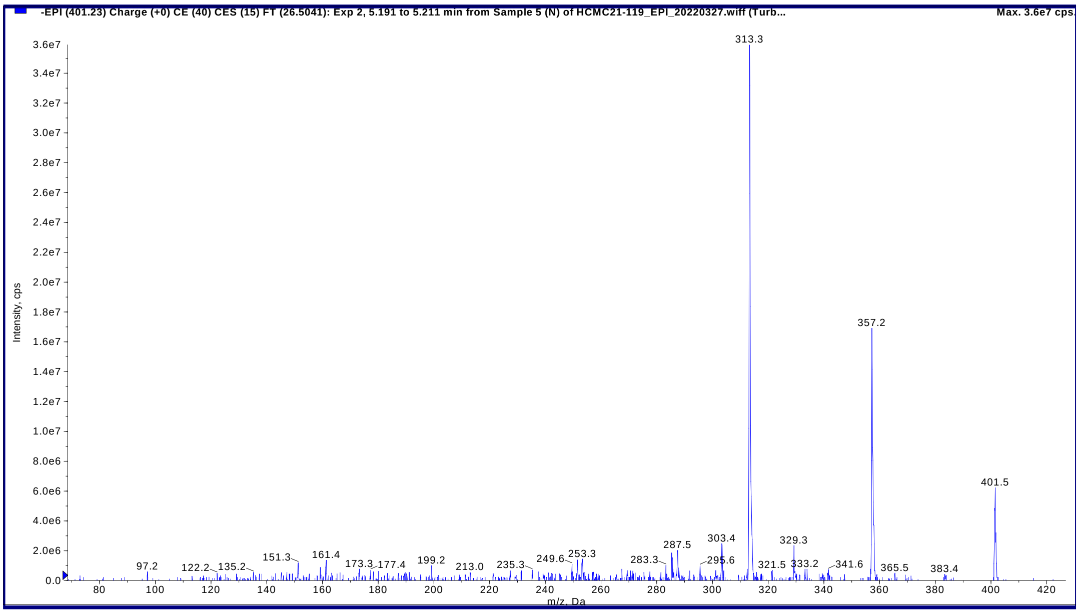

Supplement: Supplementary file 1 [file jof-08-01257-s001.zip › Supplementary Materials S4 fragmentation ions of MS spectra.pdf]
